# Effect of the Administration of Alpha-Lipoic Acid on Contrast Sensitivity in Patients with Type 1 and Type 2 Diabetes

**DOI:** 10.1155/2014/131538

**Published:** 2014-02-10

**Authors:** Anna Gębka, Ewelina Serkies-Minuth, Dorota Raczyńska

**Affiliations:** ^1^Department of Ophthalmology, Medical University of Gdańsk, Smoluchowskiego 17, 80-214 Gdańsk, Poland; ^2^Department of Anesthesiology and Intensive Care Medicine, Medical University of Gdańsk, Smoluchowskiego 17, 80-214 Gdańsk, Poland

## Abstract

The aim of this study was to estimate the effects of oral supplementation of alpha-lipoic acid (ALA) on contrast sensitivity (CS) in patients with type 1 diabetes mellitus (T1DM) and type 2 diabetes mellitus (T2DM). The study included 12 patients with T1DM aged 43±12 years, 48 patients with T2DM aged 59±10 years, and 20 control subjects aged 33±8 years. Patients from each studied group, including the control group, were randomly assigned to receive 300 mg of ALA orally once daily for 3 months. CS was evaluated with the Functional Acuity Contrast Test (FACT, Stereo Optical). In the group of patients with T1DM receiving ALA for 3 months CS remained stable and improved in those with T2DM. Reduction of CS in both T1DM and T2DM patients without alpha-lipoic acid supplementation was observed. In the control group on alpha-lipoic acid supplementation, CS improvement was noticed at one spatial frequency. Changes in the CS were observed, despite stable visual acuity and eye fundus image in all studied subjects. Our study demonstrated that oral administration of alpha-lipoic acid had influence on CS in both T1DM and T2DM patients.

## 1. Introduction

Diabetic retinopathy (DR) is a chronic and potentially sight-threatening disease resulting from microvascular damage to the retina. Oxidative stress and inflammation have been implicated in the development and progression of this diabetic ocular complication, and thus therapies intervening at the level of pathogenesis are under investigation [1, 2]. Chronic hyperglycemia, which initiates the development of DR [3, 4], generates reactive oxygen species (ROS) in the retinal tissue, characterized by high oxygen partial pressure of oxygen. ROS, mainly superoxide, inactivate glyceraldehyde-3-phosphate dehydrogenase (GADPH), an enzyme crucial in the process of glycolysis [5, 6]. This metabolic block directs substrate flux into biochemical pathways leading to endothelial cell damage. This process constitutes the unifying mechanism of hyperglycemia induced cellular damage [7].

Role of inflammation in the pathophysiology of DR has been highlighted by many researchers in many manuscripts [8–11]. The authors suggest that blood-retinal barrier damage is due to leukocytes attachment to the vascular epithelium, whereas oxidative stress, resulting in endothelial-cell dysfunction, induces the expression of adhesion molecules on the cell surface, such as vascular cell adhesion molecule-1 (VCAM-1) and intracellular adhesion molecule-1 (ICAM-1). Upregulation of these adhesion molecules appears in early DR [12]. The featured processes lead to vision impairment in patients with diabetes. It can be detected by contrast sensitivity (CS) testing, a tool more sensitive than standard visual acuity measures [13–15]. A few studies demonstrated impaired CS in patients with type 1 diabetes mellitus (T1DM) and 2 diabetes mellitus (T2DM) [14, 16].

Current efforts are aimed at therapies focused on normalizing the parameters of oxidative stress and inflammation in DR [17–19]. The beneficial effects of alpha-lipoic acid (ALA) on experimental diabetic retinopathy [20, 21] prompted us to explore the potential influence of ALA on appearance and progression of retinopathy in diabetic patients, by evaluating their CS.

ALA is a 6,8-dithio-octanoic acid and was first isolated by Reed and colleagues from bovine liver in 1950 [22]. It is an eight-carbon disulphide and contains two thiol groups. ALA, also known as thioctic acid, in vivo may be oxidized or reduced. Its reduced form, dihydrolipoic acid (DHLA), is also biologically active [23]. ALA has an asymmetric carbon, thus resulting in two isomers: R-enantiomer (R-ALA) and S-enantiomer (S-ALA). Lipoic acid supplements contain R-ALA or a racemic mixture of R-ALA and S-ALA. R-ALA is endogenously synthesized and covalently bound in proteins to the amino group of lysine, a cofactor for mitochondrial dehydrogenase enzyme complex (pyruvate dehydrogenase and alpha-ketoglutarate dehydrogenase mitochondrial enzyme complexes) [24]. Since pyruvate dehydrogenase catalyzes the oxidative decarboxylation of pyruvate to acetyl-CoA, ALA plays an essential role in pathways generating energy from glucose in mitochondria [25].

As mentioned before, oxidative stress plays an important role in the etiology of DR and antioxidants may have a great contribution in its prophylaxis and treatment. ALA fulfills criteria for an ideal antioxidant stated by Packer et al. [26]: it is absorbed from the diet, then becomes converted in cells into a usable form, and has a low toxicity and both hydrophilic and hydrophobic properties. Because of amphiphilic character of ALA, its antioxidant action takes place in the cytosol, in the plasma membrane, and in the serum and lipoproteins [24]. As an antioxidant, ALA scavenges ROS and is also able to regenerate endogenous oxidized antioxidants, such as glutathione, vitamin C, E, and coenzyme Q10. DHLA has the capacity to reduce the oxidized forms of these antioxidants and thus activates them [27].

In light of the above insights, we attempted to investigate the potential influence of oral supplementation with ALA on contrast sensitivity in patients with T1DM and T2DM.

## 2. Materials and Methods

### 2.1. Studied Subjects

Twelve patients with type 1 diabetes mellitus (8 male, 4 female; mean age 43 ± 12 years; 19 ± 12 years since diagnosis; HbA_1c_7.4 ± 1.1; 11 eyes without DR, 11 eyes with nonproliferative DR), 48 patients with type 2 diabetes mellitus (29 male, 19 female; mean age 59 ± 10 years; 7 ± 8 years since diagnosis; HbA_1c_7.2 ± 4.8; 71 eyes without DR, 12 eyes with nonproliferative DR), and the control group represented by 20 healthy people (5 male, 15 female; mean age 33 ± 8 years; 38 eyes) participated in this prospective study. Diabetes was diagnosed according to the Polish Diabetes Association guidelines which correspond with the guidelines of the American Diabetes Association [28, 29]. All studied patients underwent a complete ophthalmologic examination, including ETDRS chart visual acuity evaluation, slit-lamp biomicroscopy, and contrast sensitivity (CS) examination. Exclusion criterion was visual acuity lower than 20/25. All patients with type 1 diabetes mellitus (T1DM) had negative medical history of cardiovascular disease, diabetic neuropathy, and nephropathy, and then no other DR eye complications were observed during ophthalmoscopic examinations. Among the ones with type 2 diabetes mellitus (T2DM), 3 patients had positive medical history of cardiovascular disease and 10 of hypertension. All T2DM patients had negative medical history of renal disease and diabetic neuropathy. Moreover, in the T2DM group, 4 patients had early stages of cataract, 2 patients had glaucoma, and two of them had pseudophakia. 77% of T2DM patients were receiving oral hypoglycemic medications, 19% were on insulin therapy, and 4% were without diabetic treatment. Clinical characteristics of the studied patients with T1DM and T2DM as well as the control subjects are presented in Table 1.

Patients with T1DM and T2DM from each studied group, including the control group, were randomly assigned to receive 300 mg of ALA orally once daily for 3 months. Five of the 12 patients with T1DM (3 patients with nonproliferative DR, 2 patients without DR) received 300 mg of ALA orally once daily for 3 months. Twenty-eight patients with T2DM (2 patients with nonproliferative DR, 26 patients without DR) received 300 mg of ALA orally once daily for 3 months. In addition, fourteen of the twenty studied healthy subjects also received 300 mg of ALA orally once daily for 3 months.

This study was approved by the Ethics Committee of the Medical University of Gdańsk (NKBBN/250/2013).

### 2.2. Contrast Sensitivity Test

Contrast sensitivity (CS) was evaluated with the Functional Acuity Contrast Test (FACT, Stereo Optical; USA). This test provides presentation of sine-wave gratings of different spatial frequencies (1.5, 3, 6, 12, and 18 cycles per degree (cpd)) with a contrast-level change step corresponding to 0.15 log contrast sensitivity (logCS). Following the manufacturer's recommendation, the testing distance was 6 m for distance. An evaluation of the CS was done monocularly in all groups as a baseline examination and controlled after 3 months. The optimum additional spectacle corrections were used for distance. The CS measurements were performed under 4 chart luminance conditions (LC): 85.0 cd/m^2^, 3.0 cd/m^2^, 85 cd/m^2^ with illumination 135 lux/28 lux, and 3.0 cd/m^2^ with illumination 135 lux/28 lux. CS was analyzed first at the photopic level (85.0 cd/m^2^) and then under the mesopic level (3.0 cd/m^2^).

### 2.3. Statistical Analysis

All statistical calculations were performed using a statistical computer programme STATISTICA version 10.0. The data were checked for adherence to normal distribution by using the Shapiro-Wilk test. For the statistical comparison between groups, the Mann-Whitney *U* test was used. Differences with *P* value less than 0.05 were considered statistically significant.

## 3. Results

### 3.1. Subjects' Clinical Characteristics

Clinical characteristics of the studied patients with T1DM and T2DM as well as the control subjects are presented in Table 1. The study included 12 T1DM patients aged 43 ± 12 years and the mean duration of the disease was 19 ± 12 years. In this study group, there were 12 eyes without DR and 12 eyes with nonproliferative DR. In addition, we also examined 48 patients with type 2 diabetes mellitus aged 59 ± 10 years, 7 ± 8 years since diagnosis. In this group, there were 71 eyes without DR and 12 eyes with nonproliferative DR and the control group was represented by 20 healthy people aged 33 ± 8 years; 38 eyes participated in this prospective study. In the tested group, 5 patients with T1DM were receiving ALA supplementation in the dose of 300 mg once daily for 3 months. 28 patients with T2DM as well as 14 healthy controls were also receiving 300 mg of ALA orally once daily for 3 months (Table 1).

### 3.2. Effect of Oral Supplementation ALA on CS in Patients with T1DM

In patients with T1DM who in the tested group did not receive ALA supplementation, there was statistically significantly lower contrast sensitivity reported (6.5 ± 1.5 versus 6.0 ± 1.6, *P* = 0.009; 6.0 ± 1.4 versus 5.5 ± 1.6, *P* = 0.010; 5.3 ± 2.2 versus 4.8 ± 2.0, *P* = 0.005; 3.6 ± 2.6 versus 3.1 ± 2.4, *P* = 0.020) with spatial frequencies A-1.5, B-3, C-6, and D-12 cpd, respectively. However, no statistically significant difference in contrast sensitivity (5.6 ± 1.1 versus 5.6 ± 1.2, *P* = 0.614; 5.1 ± 1.7 versus 5.0 ± 1.6, *P* = 0.77; 4.2 ± 2.2 versus 4.2 ± 2.2, *P* = 0.947; 2.9 ± 2.3 versus 2.7 ± 2.3, *P* = 0.626; 1.7 ± 1.9 versus 1.5 ± 1.9, *P* = 0.365) at the tested spatial frequencies of A-1.5, B-3, C-6, D-12, and E-16 cpd was seen in T1DM patients who had received ALA supplementation for three months as compared with the baseline (Table 2).

### 3.3. Effect of Oral Supplementation ALA on CS in Patients with T2DM

In the study group of patients with T2DM, who were not supplemented with ALA at the dose of 300 mg once daily, the reported contrast sensitivity was significantly lower (5.7 ± 1.7 versus 5.3 ± 1.7, *P* = 0.000; 5.0 ± 1.8 versus 4.7 ± 1.9, *P* = 0.001; 3.9 ± 2.3 versus 3.5 ± 2.2, *P* = 0.001) at the spatial frequency of A-1.5, B-3, and C-6 cpd, respectively. However, no statistically significant difference in contrast sensitivity was shown (2.1 ± 2.3 versus 2.1 ± 2.1, *P* = 0.866, and 1.2 ± 1.9 versus 1.1 ± 1.8, *P* = 0.329) at the tested spatial frequencies of D-12 and E-16 cpd, respectively, in T2DM patients who had not received ALA supplementation in the dose of 300 mg once daily for three months, as compared with the baseline. On the other hand, in T2DM patients who had received ALA supplementation in the dose of 300 mg once daily for three months, contrast sensitivity had improved significantly (2.7 ± 2.2 versus 3.0 ± 2.2, *P* = 0.001, and 1.3 ± 2.0 versus 1.6 ± 2.0, *P* = 0.013), respectively, at the spatial frequencies of D-12 and E-16 cpd. While examining T2DM patients who had received ALA supplementation for three months, we have not though observed any statistically significant difference in contrast sensitivity (5.8 ± 1.6 versus 5.7 ± 1.5, *P* = 0.451, 5.2 ± 1.7 versus 5.3 ± 1.6, *P* = 0.737, and 4.3 ± 2.3 versus 4.4 ± 2.1, *P* = 0.377) respectively, at the frequencies of A-1.5, B-3, and C-6 cpd, as compared with the baseline (Table 3).

### 3.4. Effect of Oral Supplementation ALA on CS in Healthy Control Subjects

In the control group of healthy volunteers who had received ALA supplementation in the dose of 300 mg once daily for three months, a statistically significant contrast sensitivity improvement had been observed only at the spatial frequency B-3 (6.1 ± 1.5 versus 6.4 ± 1.2, *P* = 0.027) as compared with the baseline. However, in the healthy control group, no statistical significance had been obtained at other tested frequencies after three months as compared with the baseline, regardless of whether the participants had received ALA supplementation in the dose of 300 mg once daily (Table 4).

## 4. Discussion

So far, there has been little research that would evaluate the effects of oral treatment of patients with ALA for the help on CS in patients with T1DM and T2DM. Therefore, the aim of this study was to estimate the effect of oral supplementation with ALA on CS in patients with T1DM and T2DM. In the group of patients with T1DM receiving ALA for 3 months CS remained stable. However, in the group of patients with T1DM without ALA supplementation, significant deterioration of CS at spatial frequencies A-1.5, B-3, C-6, and D-12 cpd was observed. On the other hand, in T2DM patients on ALA supplementation CS improved after 3 months at spatial frequencies D-12 and E-18 cpd, whereas the group of T2DM patients not receiving ALA had a significant CS reduction at spatial frequencies A-1.5, B-3 and C-6 cpd. Previous sparse studies have shown that ALA and DHLA play a very important role in the treatment of microvascular dysfunction in patients with diabetes [17–19]. Du et al. showed that oral treatment with ALA combined with benfotiamine (synthetic vitamin B1) normalized increased AGE formation and reduced hexosamine pathway activity and prostacyclin synthesis in patients with type 1 DM [19]. In addition, Lin et al. examined the effect of ALA (R-enantiomer) at 60 mg/kg dose i.p. (5 days per week for 30 weeks) on diabetic rats with experimental DR [20]. Authors showed that after this treatment the number of acellular capillaries was significantly reduced and pericyte loss was inhibited. Moreover, they presented the evidence that ALA reduces oxidative stress, normalizes increased NF kappa B, AGE, and RAGE, and reduces VEGF upregulation by 43% [20]. According to Kowluru and Odenbach, long-term oral administration of ALA (400 mg/kg for 11 months) in diabetic rats inhibits capillary cell apoptosis and also reduces the number of acellular capillaries in the retina [30].

Currently, ophthalmologists have a great diagnostic tool for detection of vision impairment through contrast sensitivity test, a more sensitive instrument than standard visual acuity measures [14, 15]. In our control group, CS remained stable among the patients without ALA supplementation, while a significant CS improvement after 3-month ALA supplementation at spatial frequency 3 cpd was noticed. During our 3-month study, all studied subjects had stable visual acuity and eye fundus image; however, changes in the CS were observed. Moreover, CS improvement after ALA supplementation appeared only in the T2DM group, whether it had no influence on CS among the T1DM patients. We suggest that ALA supplementation has improved insulin sensitivity in patients with T2DM. Other clinical studies, carried out in patients with type 2 DM, provided evidence that both intravenous and oral treatment with ALA improve insulin-stimulated glucose disposal [31, 32]. Moreover, Bucolo et al. demonstrated that the fortified extract of red berries, *Ginkgo biloba,* and white willow bark containing L-carnosine and ALA may blunt some of the negative effects due to hyperglycemia, such as inflammation, oxidation, and VEGF expression in early retinal and plasma changes of diabetic rats [21].

Our research has shown that oral 3-month supplementation with ALA at a relatively low 300 mg and convenient once daily dose maintains functional vision in T1DM patients and improves it in T2DM patients. Concurrently, reduction of CS in both patients with T1DM and T2DM without ALA supplementation was observed. In the control group on ALA supplementation, CS improvement was noticed at one spatial frequency.

In summary, our results suggest that supplementation with ALA represents an achievable adjunct therapy to help prevent loss of vision in diabetic patients. Further investigations are needed to evaluate the influence of oral supplementation of ALA in patients with T1DM and T2DM.

## Figures and Tables

**Table 1:** Clinical characteristics of patients with T1DM, T2DM, and healthy control subjects.

	Age (years)	Duration of diabetes (years)	HbA1c (%)	Insulin therapy (%)	Oral hypoglycemic medications (%)	Without diabetic treatment (*N*)	Oral ALA supplementation (*N*)	Complications (*N*)
T1DM patients *N* = 12	43 ± 12	19 ± 12	7.4 ± 1.1	100	—	—	5	6 with NPDR
T2DM patients *N* = 48	59 ± 10	7 ± 8	7.2 ± 4.8	19	77	4	28	7 NPDR 4 ischemic heart disease 10 hypertension 4 cataract 2 glaucoma 2 pseudophakia
Healthy control subjects *N* = 20	33 ± 8	—	—	—	—	—	14	—

T1DM: diabetes mellitus type 1; T2DM: diabetes mellitus type 2; *N*: the number of patients.

**Table 2:** Characteristics of contrast sensitivity examinations in T1DM patients with and without ALA supplementation at baseline and after 3 months.

Spatial frequencies	T1DM patients without ALA supplementation CS × LC = 48 measurements			T1DM patients with ALA supplementation CS × LC = 40 measurements		
	Baseline	After 3 months	*P* value	Baseline	After 3 months	*P* value
A-1.5 cpd						
Mean. ± SD	6.5 ± 1.5	6.0 ± 1.6		5.6 ± 1.1	5.6 ± 1.2	
Range	3.0–9.0	3.0–9.0	*** *** *P* = 0.009**	4.0–8.0	3.0–8.0	*P* = 0.614
Median	7.0	6.0		5.0	5.0	
95% CI	[6.1; 7.0]	[5.5; 6.4]		[5.3; 6.0]	[5.2; 5.9]	

B-3 cpd						
Mean. ± SD	6.0 ± 1.4	5.5 ± 1.6		5.1 ± 1.7	5.0 ± 1.6	
Range	2.0–8.0	1.0–9.0	*** *** *P* = 0.010**	2.0–8.0	1.0–9.0	*P* = 0.770
Median	6.0	6.0		5.0	5.0	
95% CI	[5.6; 6.5]	[5.1; 6.0]		[4.5; 5.6]	[4.5; 5.5]	

C-6 cpd						
Mean. ± SD	5.3 ± 2.2	4.8 ± 2.0		4.2 ± 2.2	4.2 ± 2.2	
Range	0.0–8.0	0.0–8.0	*** *** *P* = 0.005**	0.0–8.0	0.0–8.0	*P* = 0.947
Median	6.0	5.0		4.0	4.0	
95% CI	[4.6; 5.9]	[4.2; 5.3]		[3.5; 4.9]	[3.5; 4.9]	

D-12 cpd						
Mean. ± SD	3.6 ± 2.6	3.1 ± 2.4		2.9 ± 2.3	2.7 ± 2.3	
Range	0.0–8.0	0.0–8.0	** ** *P* = 0.020**	0.0–7.0	0.0–7.0	*P* = 0.626
Median	4.0	4.0		3.0	2.0	
95% CI	[2.8; 4.4]	[2.4; 3.8]		[2.1; 3.6]	[2.0; 3.5]	

E-18 cpd						
Mean. ± SD	2.1 ± 2.2	2.0 ± 2.0		1.7 ± 1.9	1.5 ± 1.9	
Range	0.0–7.0	0.0–5.0	*P* = 0.530	0.0–5.0	0.0–6.0	*P* = 0.365
Median	1.0	1.5		0.5	0.0	
95% CI	[1.4; 2.7]	[1.4; 2.5]		[1.1; 2.3]	[0.8; 2.1]	

CS × LC: the number of contrast sensitivity measurements of each eye in 4 luminance conditions and under 5 spatial frequencies.

A-1.5, B-3, C-6, D-12, and E-18 cpd: spatial frequencies.

**Statistically significant differences between the baseline examination of T1DM patients without ALA supplementation versus after 3 months.

**Table 3:** Characteristics of contrast sensitivity examinations in T2DM patients with and without ALA supplementation at baseline and after 3 months.

Spatial frequencies	T2DM patients without ALA supplementation CS × LC = 132 measurements			T2DM patients with ALA supplementation CS × LC = 200 measurements		
	Baseline	After 3 months	*P* value	Baseline	After 3 months	*P* value
A-1.5 cpd						
Mean. ± SD	5.7 ± 1.7	5.3 ± 1.7		5.8 ± 1.6	5.7 ± 1.5	
Range	1.0–9.0	0.0–9.0	*** *** *P* = 0.000**	0.0–9.0	1.0–9.0	*P* = 0.451
Median	6.0	5.0		6.0	6.0	
95% CI	[5.4; 6.0]	[5.0; 5.5]		[5.6; 6.0]	[5.5; 5.9]	

B-3 cpd						
Mean. ± SD	5.0 ± 1.8	4.7 ± 1.9		5.2 ± 1.7	5.3 ± 1.6	
Range	0.0–8.0	0.0–9.0	*** *** *P* = 0.001**	0.0–8.0	0.0–9.0	*P* = 0.7371
Median	5.0	5.0		5.0	5.0	
95% CI	[4.7; 5.4]	[4.3; 5.0]		[5.0; 5.5]	[5.0; 5.5]	

C-6 cpd						
Mean. ± SD	3.9 ± 2.3	3.5 ± 2.2		4.3 ± 2.3	4.4 ± 2.1	
Range	0.0–9.0	0.0–8.0	*** *** *P* = 0.001**	0.0–9.0	0.0–9.0	*P* = 0.377
Median	4.0	4.0		5.0	4.5	
95% CI	[3.5; 4.3]	[3.1; 3.9]		[3.9; 4.6]	[4.1; 4.7]	

D-12 cpd						
Mean. ± SD	2.1 ± 2.3	2.1 ± 2.1		2.7 ± 2.2	3.0 ± 2.2	
Range	0.0–7.0	0.0–7.0	*P* = 0.866	0.0–8.0	0.0–9.0	*** *** *P* = 0.001*
Median	1.0	2.0		3.0	3.0	
95% CI	[1.7; 2.5]	[1.7; 2.5]		[2.3; 3.0]	[2.7; 3.3]	

E-18 cpd						
Mean. ± SD	1.2 ± 1.9	1.1 ± 1.8		1.3 ± 2.0	1.6 ± 2.0	
Range	0.0–7.0	0.0–7.0	*P* = 0.329	0.0–9.0	0.0–8.0	*** *** *P* = 0.013*
Median	0.0	0.0		0.0	0.0	
95% CI	[0.9; 1.5]	[0.8; 1.4]		[1.0; 1.6]	[1.3; 1.9]	

CS × LC: the number of contrast sensitivity measurements of each eye in 4 luminance conditions and under 5 spatial frequencies.

A-1.5, B-3, C-6, D-12, and E-18 cpd: spatial frequencies.

*Statistically significant differences between the baseline examination of T2DM patients with ALA supplementation versus after 3 months.

**Statistically significant differences between the baseline examination of T2DM patients without ALA supplementation versus after 3 months.

**Table 4:** Characteristics of contrast sensitivity examinations in the control group with and without ALA supplementation at baseline and after 3 months.

Spatial frequencies	Control group without ALA supplementation CS × LC = 44 measurements			Control group with ALA supplementation CS × LC = 108 measurements		
	Baseline	After 3 months	*P* value	Baseline	After 3 months	*P* value
A-1.5 cpd						
Mean. ± SD	6.9 ± 1.6	6.7 ± 1.3		6.7 ± 1.6	6.8 ± 1.2	
Range	4.0–9.0	5.0–9.0	*P* = 0.318	0.0–9.0	5.0–9.0	*P* = 0.465
Median	7.0	7.0		7.0	7.0	
95% CI	[6.4; 7.4]	[6.3; 7.1]		[6.4; 7.0]	[6.6; 7.1]	

B-3 cpd						
Mean. ± SD	6.8 ± 1.6	6.7 ± 1.6		6.1 ± 1.5	6.4 ± 1.2	
Range	3.0–9.0	3.0–9.0	*P* = 0.423	0.0–9.0	3.0–9.0	*** *** *P* = 0.027*
Median	6.5	7.0		6.0	7.0	
95% CI	[6.3; 7.3]	[6.2; 7.2]		[5.8; 6.4]	[6.2; 6.7]	

C-6 cpd						
Mean. ± SD	5.9 ± 1.8	6.0 ± 1.5		5.4 ± 1.9	5.5 ± 1.7	
Range	1.0–9.0	2.0–8.0	*P* = 0.414	0.0–8.0	0.0–8.0	*P* = 0.399
Median	6.0	6.0		5.0	6.0	
95% CI	[5.3; 6.4]	[5.5; 6.4]		[5.1; 5.8]	[5.2; 5.8]	

D-12 cpd						
Mean. ± SD	4.4 ± 2.3	4.5 ± 2.2		4.3 ± 1.9	4.2 ± 1.9	
Range	0.0–9.0	0.0–9.0	*P* = 0.648	0.0–8.0	0.0–8.0	*P* = 0.446
Median	4.0	4.5		4.0	4.0	
95% CI	[3.7; 5.1]	[3.8; 5.2]		[3.9; 4.7]	[3.8; 4.5]	

E-18 cpd						
Mean. ± SD	2.9 ± 2.6	2.5 ± 2.4		2.9 ± 2.2	2.6 ± 1.9	
Range	0.0–9.0	0.0–8.0	*P* = 0.127	0.0–9.0	0.0–7.0	*P* = 0.234
Median	3.0	2.0		3.0	2.0	
95% CI	[2.1; 3.7]	[1.8; 3.3]		[2.5; 3.3]	[2.3; 3.0]	

CS × LC: the number of contrast sensitivity measurements of each eye in 4 luminance conditions and under 5 spatial frequencies.

Spatial frequencies: A-1.5, B-3, C-6, D-12, and E-18 cpd.

*Statistically significant differences between the baseline examination of control group with ALA supplementation versus after 3 months.
